# The most important questions in cancer research and clinical oncology: Question 1. Could the vertical transmission of human papilloma virus (HPV) infection account for the cause, characteristics, and epidemiology of HPV-positive oropharyngeal carcinoma, non-smoking East Asian female lung adenocarcinoma, and/or East Asian triple-negative breast carcinoma?

**DOI:** 10.1186/s40880-016-0168-1

**Published:** 2017-01-16

**Authors:** Joseph T. S. Wee, Sharon Shuxian Poh

**Affiliations:** 1 Division of Radiation Oncology, National Cancer Centre, Singapore, 169610 Singapore; 2 Duke-NUS Medical School, Singapore, 169857 Singapore

## Abstract

Specific research foci: (1) Mouse models of gamma-herpes virus-68 (γHV-68) and polyomavirus (PyV) infections during neonatal versus adult life. (2) For human papilloma virus (HPV)-positive oropharyngeal carcinoma (OPC)—(a) Asking the question: Is oral sex a powerful carcinogen? (b) Examining the evidence for the vertical transmission of HPV infection. (c) Examining the relationship between HPV and Epstein–Barr virus (EBV) infections and nasopharyngeal cancer (NPC) in West European, East European, and East Asian countries. (d) Examining the association between HPV-positive OPC and human leukocyte antigen (HLA). (3) For non-smoking East Asian female lung adenocarcinoma—(a) Examining the incidence trends of HPV-positive OPC and female lung adenocarcinoma according to birth cohorts. (b) Examining the association between female lung adenocarcinoma and HPV. (c) Examining the associations of lung adenocarcinoma with immune modulating factors. (4) For triple-negative breast carcinoma (TNBC) in East Asians—(a) Examining the association between TNBC and HPV. (b) Examining the unique epidemiological characteristics of patients with TNBC. A summary “epidemiological” model tying some of these findings together.

## Mouse model

Mice infected with different viruses during neonatal or adult life display different outcomes.

For PyV infection, Rowe et al. [1] first reported that “only infection of new-born mice resulted in persistently infected mice that were subsequently able to transmit PyV infection to other mice, whereas mice infected as adults were unable to transmit PyV infections or establish persistent infections.” Immunocompetent adult mice do not appear to be affected by PyV infection. Demengeot et al. [2] showed that infection is persistent in some epithelial tissues (the skin, mammary and salivary glands), lymphoid organs (the spleen and nodes), and mesenchymal bone tissue.

For γHV-68 infection, Ptaschinski and Rochford [3] observed that unlike infection of adult mice, infection of 8-day-old pup mice with γHV-68 results in disseminated acute infection, delayed clearance and persistence of the virus in the lungs, and no significant infectious mononucleosis-like syndrome.

Ptaschinski and Rochford [3] also noted that infection with other viruses, such as the respiratory syncytial virus, hepatitis viruses, and cytomegalovirus, at a young age are all associated with chronic infection, and this may be “due to differences in the infant and adult immune systems.” In hepatitis occurring in woodchucks, chronic persistence may be “due in part to a decrease in the T cell response along with a decrease in Th1-associated cytokines.” Similarly, children infected with cytomegalovirus exhibit a decreased interferon-γ production and decreased CD4^+^ T-cell response.

Ptaschinski and Rochford [3] further commented that while most children in developing countries are infected with EBV by the age of 1, most studies on EBV pathogenesis rely on cells isolated from either asymptomatic adults or adults with infectious mononucleosis. Burkitt’s lymphomas occur primarily in childhood and usually present in the jaw area, and it has been hypothesized that early EBV infection is a risk factor. Ptaschinski and Rochford [3] mentioned that “whether early age of infection leads to higher viral persistence in mucosal sites in children remains to be determined” and potentially “γHV-68 infection of young mice can be used as a model to study age-dependent persistence of γHV-68 infection at mucosal sites.”

Toll-like receptor 8 (TLR8) is involved in the innate immune response to different pathogens. In mice, TLR8 lacks five amino acids and is non-functional and perhaps redundant, but it plays a critical role in humans as it is the only TLR that is active in the neonatal period as described by Levy and Zarember [4]. Cheng et al. [5] showed that the allele frequencies of TLR8 in East Asians significantly differed from those in African–Americans and Caucasians and that it may be an important factor in the disparity of viral infections amongst different ethnic groups. Barreiro et al. [6] further showed that the “human TLR8 is the TLR under the strongest purifying selection.”

## HPV-positive OPC

### Is oral sex a powerful carcinogen?

Genden et al. [7] wrote that although HPV-positive oropharyngeal squamous cell cancer has been linked to sexual practices and increasing numbers of sexual partners, it is also present in many individuals reporting few sexual partners. Only a small percentage of individuals have a high number of partners, and a small number of sexual partners do not lower the risk. Moreover, HPV-positive oropharyngeal squamous cell cancer appears to affect men more than women.

In a provocative article, Rosenquist [8] summarized the data for cervical cancer and noted that the majority of HPV-infected women were able to clear their cervico-vaginal infections spontaneously, leaving only a small percentage subsequently developing cervical cancer; thus making HPV infection a necessary, but not the sole factor, in the development of cervical cancer. She further highlighted several studies that showed that cervico-vaginal HPV infection is predominantly self-limiting with a median duration of 8–12 months. In large epidemiological studies, “the risk factors most consistently associated with progression of high-risk HPV to cervical cancer were as follows: high parity (number of children), long-term oral contraceptive use, smoking, and co-infection with other sexually transmitted diseases” [8].

In a longitudinal prospective Finnish family HPV infection study [9] highlighted by Rosenquist to question the role of oral sex in HPV infection transmission, couples were tested at both oral and genital sites over several occasions. It was found that “the oral and genital HPV types found in couples differ so that the strains infecting one partner are only modestly correlated with strains infecting the other, with the prevalence of oral high-risk HPV infections fluctuating between 15% and 27%. Oral and genital HPV strains were not only poorly correlated within subjects but also highly discordant within couples. 12% of women and 26% of men reported regular oral sex. However, oral sex habits showed no association with oral or genital HPV infection. Similarly, no association was established between oral HPV infections in either spouse and the outcome of the partner’s genital HPV infection.” Conversely, the researchers noted that “a persistent oral HPV infection of the spouse increased the risk of persistent oral HPV infection tenfold in the other spouse” [9]. Also notable was that men tended to begin and complete clearance of HPV infections earlier than women. This study thus implies the importance of persistent oral infection over the mere presence of infection or the practice of oral sex in contributing to the correlation of HPV within a couple.

A literature review on the sexual transmission of virus infections by Edwards and Carne [10] in 1998 concluded that “the evidence favouring oral transmission of HPV is both based on case studies, and is only correlational evidence.” Rosenquist [8] further highlights a large survey (*n* = 12,571) done in 2002 by the United States Centers for Disease Control and Prevention, showing that cunnilingus was practiced by 90% of male respondents and fellatio by 88% of female respondents. “Similarly, research from other cultures shows oral sexual contact to be very common, and oral genital contact is more common than anal intercourse among gay and bisexual men.” Thus, the “link between oral sex, HPV and cancer is not a simple cause and effect relationship,” especially as oral sex behaviors appear to be common and widespread, thus making any perceived relationship not significant. This led Rosenquist to conclude that many factors are involved in making an HPV infection to become persistent, and subsequently, to become carcinogenic; some are unknown, and others are likely involved in compromises to the immune system.

In a meta-analysis, Li et al. [11] demonstrated that “oral sex is only a risk marker rather than an independent risk factor for oral cancer.” On the other hand, Zeng et al. [12, 13] demonstrated that both tooth loss and periodontal disease are independent risk factors for head and neck cancers. Similarly, Tezal et al. [14] reported that the tumor HPV infection status of patients with head and neck cancers may be associated with a history of chronic inflammatory disease in the oral cavity, with this association being the strongest amongst OPC patients.

One might be tempted to hypothesize that having multiple sexual partners might be an indicator for chronic infection and inflammation associated with sexual transmission of HPV and other virus infections, which might have the same effect as chronic periodontitis perhaps through the systemic bystander effects of the immune system reacting to chronic infection and inflammation.

### Evidence for the vertical transmission of HPV infection

In a review article, Mammas et al. [15] stated that “HPV can be transmitted through physical contact, as well as vertically from the HPV-positive mother to her new-born and can cause subclinical or clinical infections. HPV-associated clinical infections include genital warts, skin warts, recurrent respiratory papillomatosis, squamous intraepithelial lesions and cervical cancer.” A recent review by Syrjänen [16] suggests that vertical transmission of HPV infection occurs in approximately 20% of cases. HPV infections in the oral mucosa of infants are silent infections and are found in approximately 10% of infants. According to the review by Syrjanen [16], the concordance of HPV infections in the oral and genital specimens of mothers and their new-borns ranges from 0.2% to 73%. The true impact of the silent infection of HPVs in childhood remains to be further investigated. On the other hand, the viral loads for persistent infection may be low and escape current detection techniques [17]. In similar light, it has been difficult to identify persistent EBV infection in epithelial cells; however, circumstantial evidence points toward early infection. In addition, a few studies isolated the EBV genome from the parotid gland as well as the virus from the ducts and orifices of the parotid glands [18].

### Relationship between HPV and EBV

Several studies suggest that EBV and HPV infections do not co-exist in NPC patients in West and East European countries [19, 20].

Early EBV infection appears to be related to socio-economic status and demonstrates a decreasing trend from East Asia through East to West Europe. On the other hand, the incidence trend of adolescent EBV infection in the form of infectious mononucleosis is reversed, and this effect is also reflected by the incidence of multiple sclerosis. Of interest, the male-to-female ratio for multiple sclerosis is the highest in East Asia and gradually decreases as one moves west, which supports the hypothesis that early EBV infection predisposing East Asians to NPC could be related to a sex-linked recessive form of transmission [21].

HPV-positive OPC follows a reverse trend, with male Westerners exhibiting the highest incidences. The incidences gradually decrease as one moves east. This result is a “perfect” mirror image of EBV-related NPC. On the other hand, although HPV-positive OPC is rare in East Asian females, 60% of these females who develop OPC are HPV-positive [22] (Table 1).

**Table 1:** Selected clinical characteristics of EBV and HPV infections across West Europe, East Europe, and East Asia

Clinical characteristic	West Europe	East Europe	East Asia
Early EBV infection	+	++	++++
Late EBV infection: infectious mononucleosis (IM)	++++	++	+
Male-to-female ratio (IM)	1:1.5	Not available	Not available
Prevalence of (hypothesized late EBV infection) multiple sclerosis (MS) (per 10^5^)	>30	5–30	<5
Male-to-female ratio (MS)	1:2	Not available	1:5
Hypothesized early infection with EBV in males	+	++	++++
Hypothesized early infection with EBV in females	Rare	+	++
Hypothesized early infection with HPV in males	++++	++	+
Hypothesized early infection with HPV in females	+++	++	+++
Proportion of patients with HPV-positive OPC (%)	66	30	10
Proportion of female OPC patients who are HPV-positive (%)	45	40	60
Proportion of male OPC patients who are HPV-positive (%)	65	40	30
Male-to-female ratio	1.5	1.0	0.5
Socio-economic status (1950s–1960s)	++++	++	+
Associated chronic infections (e.g., chronic periodontitis and tuberculosis)	+	+++	++++
Male HPV-positive OPC	++++	+	Rare
Female nasopharyngeal carcinoma	Rare	+	++
Male nasopharyngeal carcinoma	+	++	++++
Female non-smoking lung adenocarcinoma	+	++	++++
Triple-negative breast cancer	+	++	+++

*EBV* Epstein–Barr virus, *HPV* human papilloma virus, *OPC* oropharyngeal carcinoma

One potential explanation of why EBV and HPV are “complementary” but do not usually co-exist is that both EBV and HPV infections could conceivably persist in salivary gland tissues. Vestigial ectopic salivary gland tissues can be found in human tonsils and tongue base [23], and this may account for the predilection of HPV-related cancers in these sites compared with other head and neck sites. Of note, EBV-related Eskimomas and other non-NPC lymphoepitheliomas can also be found in other head and neck sites and the lungs.

Finally, the sexual revolution, which started just after the Second World War, puts the 1950s birth cohort at risk. If these individuals were to develop cancers 40 years later (after vertical transmission of HPV infection), HPV-positive OPC would be expected to become evident only after the 1990s, which would fit into the epidemiological picture of this cancer.

### Association between HPV-positive OPC and HLA

A recent genome-wide association study (GWAS) [24] showed that “oropharyngeal cancer associations were limited to the human leukocyte antigen (HLA) region, and this association was considerably stronger in HPV-positive cancers.” What is more intriguing is that HLA again appears to play an important and consistent role in several other virus-related cancers, such as NPC [25], extra-nodal natural killer T-cell lymphoma [26], EBV-positive Hodgkin’s lymphoma [27], and chronic hepatitis B-associated hepatocellular carcinoma [28].

## Non-smoking East Asian female lung adenocarcinoma

### Birth cohort

Interestingly, it was the 1950s birth cohort that exhibited a spike in female lung adenocarcinoma in Japan [29] and Hong Kong [30], and this pattern is intriguingly similar to that for HPV-positive OPC.

### Associations between female lung adenocarcinoma and HPV

Anatharaman et al. [31] observed a significantly increased risk of lung cancer in the presence of various HPV antibodies. Similar effects were observed for never, former, and current smokers. These results indicate that HPV antibodies are substantially increased in lung cancer patients compared with hospital-based controls, none of whom reported a previous cancer history or any tobacco-related disease. They subsequently reported that “although nearly 10% of the lung tumours were positive for any HPV DNA (7% for HPV16 DNA), none expressed the viral oncogenes” [32]. It should however be noted that all these studies were performed in Europe.

On the other hand, an East Asian study showed a significant increase in lung cancer risk amongst Taiwanese women with HPV infection [33]. An “adaptive” meta-analysis also revealed a significant effect of HPV infection in lung cancer risk in East Asian women and never smokers [34].

### Associations between female lung adenocarcinoma and immune-modulating factors

Associations are noted between lung adenocarcinoma and HLA [35], chronic rhinosinusitis [36, 37], and latent pulmonary tuberculosis [38]. In a Japanese study, “a decreased number of remaining teeth was associated with increased OR (odds ratio) of lung cancer [OR 1.54; 95% CI (confidence interval) 1.05–2.27; *P* trend = 0.027]” [39]. Two studies looking specifically at lung adenocarcinomas in Japanese also showed that HLA class II at 6p21.32 was associated with epidermal growth factor receptor (EGFR) mutation-positive lung adenocarcinoma [40] and that major histocompatibility complex, class II, DM alpha (HLA-DMA) was overexpressed in female Japanese patients compared with male Japanese patients [41].

In sheep, the Jaagsiekte sheep retrovirus (JSRV), which can induce neoplastic transformation of the alveolar and bronchiolar secretory epithelial cells, is the cause of ovine pulmonary adenocarcinoma [42].

## Triple-negative breast carcinoma in East Asians

### Associations between TNBC and HPV

Lawson et al. [43] observed “the presence of high-risk HPV gene sequences in breast cancers; that HPV oncogenic influences may occur early in the development of breast cancer; that HPVs in breast cancer are likely to be biologically active (as shown by transcription of HPV DNA to RNA plus the expression of HPV E7 proteins), and that there appeared to be a correlation between high-risk HPV in benign breast specimens and subsequent HPV-positive breast cancer in the same patient.” In another study, Subhawong et al. [44] showed that basal-like breast carcinomas, which resemble HPV-associated squamous cell carcinomas morphologically, frequently exhibit a similar Rb^−^/p16^+^ phenotype. A study from Singapore showed a high prevalence of infection with high-risk HPV in breast cancer patients [45]. A Mexican study detected high-risk HPV DNA in 8 (40.0%) of 20 metaplastic breast carcinomas [7 (87.5%) with HPV-16 infection and 1 (12.5%) with HPV-18 infection)] [46]. Another study from China, using paraffin-embedded breast cancer tissue samples, detected HPV18 E6 and HPV18 E7 in approximately 30% of the samples [47].

In a meta-analysis of case–controlled studies, HPV infection was found to have increased the risk of breast cancer [summary odds ratio (SOR) =  4.02, 95% CI 2.42–6.68] [48]. In another meta-analysis, Li et al. [49] showed that 25% of the breast cancers were associated with HPV infection, of which approximately 30% occurred in Asia and 13% in Europe.

Recent studies also showed that “there were more cases with high expressions of HLA-G in non-luminal than in luminal subtypes (of breast cancer)” [50] and that “HLA expression was inversely correlated with oestrogen receptor (ER) expression in normal and cancerous breast tissue” [51].

Another study identified that the gene *Lgr6*, which labels progenitor cells, was associated with the luminal type of breast tumors [52]. One might be tempted to hypothesize that if a viral infection had hijacked the stem cell, the result might well be a non-luminal type of breast tumor. Intriguingly, two separate GWAS studies revealed that an *Lgr6* single nucleotide polymorphism was associated with estrogen receptor (ER)-negative breast cancer [53], and the gene was also associated with TNBC [54]. It is also noteworthy that “the murine MMTV-Wnt1 model of mammary cancer shares transcriptional patterns with, and exhibits hallmarks of, human triple-negative breast cancer (TNBC)” [55].

### Epidemiology of TNBCs

Younger women, who are diagnosed with breast cancer, appear to have more aggressive tumors and a higher incidence of poorly differentiated (grade III) and ER/progesterone receptor (PR)-negative cancers compared with older women. Interestingly, a study from Malaysia showed that TNBC appears to be associated with a lower socio-economic status and that TNBC was most common in the native population of Sarawak (37%) and less frequent in Chinese (23%) and Malays (33%) [56]. Similar high incidences of TNBC were noted in the women of Northeast India [57] and Indonesia [58].

A recent study assessing breast cancer incidence trends in Japan [59] exhibited an increased incidence in young patients around the 1980s–1990s period, again reflecting a similar pattern to both HPV-positive OPC and non-smoking East Asian female lung adenocarcinoma. A similar phenomenon was also observed in another study from Taiwan, China [60].

A study from Sweden reported an increased incidence of breast cancer within 15 years, amongst the women who were diagnosed with periodontal disease at an initial examination [61]. The average age of the women was 46 years.

## Summary and “epidemiological” model

Although no definite cause and association can be concluded from the above, the circumstantial evidence suggests the plausibility of the vertical transmission of HPV infection giving rise to HPV-positive OPC, non-smoking East Asian female lung adenocarcinoma, and East Asian TNBC (Table 1).

## Clinical implications

The following two potential clinical implications can arise:

1. If HPV infection is a causative factor and is transmitted vertically in the three types of cancers, there are potential applications in terms of
  - Prevention—using a HPV vaccination strategy applied during the perinatal period similar to that in the hepatitis B programme.
  - Targeted screening—for example, using HPV serology measured during the first month of life together with a susceptibility marker, such as “susceptible” HLA alleles.
2. The second observation is that the three types of cancers appear to be associated with
  - A favorable response to the new checkpoint inhibitors.
  - Some chronic infections/inflammation such as chronic periodontitis or chronic rhinosinusitis.

These findings might imply that the immune reaction associated with chronic infection might have a systemic bystander effect, namely affecting the body’s immune surveillance toward the pre-cancer, perhaps removing the immune suppressive effect, and thus allowing the cancer to grow [62]. It is conceivable that tackling chronic infection, such as chronic periodontitis, may reduce the incidence of such cancers [63]. Studies have demonstrated that “subgingival scaling in patients with periodontitis reduced inflammatory markers” and that the regular use of aspirin may reduce the long-term risks of colorectal, esophageal, breast, and other cancers. Perhaps periodontal disease should deserve a classification as a public health problem.
